# Association of Neoadjuvant Chemotherapy With Overall Survival in Women With Metastatic Endometrial Cancer

**DOI:** 10.1001/jamanetworkopen.2020.28612

**Published:** 2020-12-09

**Authors:** Claire J. Tobias, Ling Chen, Alexander Melamed, Caryn St Clair, Fady Khoury-Collado, Ana I. Tergas, June Y. Hou, Chin Hur, Cande V. Ananth, Alfred I. Neugut, Dawn L. Hershman, Jason D. Wright

**Affiliations:** 1 Vagelos College of Physicians and Surgeons, Columbia University, New York, New York; 2 New York-Presbyterian Hospital, New York; 3 Herbert Irving Comprehensive Cancer Center, New York, New York; 4 Joseph L. Mailman School of Public Health, Columbia University, New York, New York; 5 Rutgers Robert Wood Johnson Medical School, New Brunswick, New Jersey; 6 Environmental and Occupational Health Sciences Institute, Piscataway, New Jersey

## Abstract

**Importance:**

Although primary debulking surgery (PDS) is often considered the criterion standard for treatment of stage IV endometrial cancer, PDS is associated with significant morbidity and poor survival. Neoadjuvant chemotherapy (NACT) has been proposed as an alternative treatment strategy.

**Objective:**

To determine the use of and outcomes associated with NACT for women with stage IV endometrial cancer.

**Design, Setting, and Participants:**

This cohort study used the National Cancer Database to identify women with stage IV endometrial cancer treated from January 1, 2010, to December 31, 2015. The cohort was limited to women aged 70 years or younger with minimal comorbidity (comorbidity score = 0). Women were stratified based on receipt of NACT or PDS. A propensity score analysis with inverse probability weighting was performed to balance the clinical characteristics of the groups. Survival was examined using flexible parametric Royston-Parmer models to account for time-varying hazards associated with use of NACT. An intention-to-treat (ITT) analysis was performed, as was a per-protocol (PP) analysis that included only women who received treatment with both chemotherapy and surgery (in either sequence). Data were analyzed from March 15, 2018, to July 20, 2018.

**Main Outcomes and Measures:**

Use of NACT and overall survival.

**Results:**

Of a total of 4890 women (median age, 60 years [interquartile range, 54-65 years]) with stage IV endometrial cancer, NACT was used in 952 women (19.5%). Use of NACT increased from 106 of 661 women (16.0%; 95% CI, 13.2%-18.8%) in 2010 to 224 of 938 women (23.9%; 95% CI, 21.2%-26.6%) in 2015 (*P* < .001). In a multivariate model, more recent year of diagnosis (risk ratio [RR], 1.42; 95% CI, 1.21-1.79 for 2015 vs 2010), stage IVB disease (RR, 1.31; 95% CI, 1.03-1.67 for stage IVB vs IVA), and serous histology (RR, 1.38; 95% CI, 1.13-1.69 for serous vs endometrioid histology) were associated with use of NACT. In a propensity score–balanced cohort, use of NACT displayed a time-varying association with survival. In the ITT analysis, use of NACT was associated with decreased mortality for the first 3 months after diagnosis (hazard ratio [HR] at 2 months, 0.81; 95% CI, 0.66-0.99). After 4 months, the survival curves crossed, and receipt of NACT was associated with increased mortality (HR at 6 months, 1.23; 95% CI, 1.09-1.39). In the PP analysis, use of NACT was associated with decreased mortality for the first 8 months after diagnosis (HR at 6 months, 0.79; 95% CI, 0.63-0.98). After 9 months, the survival curves crossed, and receipt of NACT was associated with increased mortality (HR at 12 months, 1.22; 95% CI, 1.04-1.43).

**Conclusions and Relevance:**

The results of this cohort study suggest that women treated with PDS are at increased risk of early death but have a more favorable long-term prognosis. In contrast, results suggest that women treated with NACT, particularly if they ultimately undergo surgery, may have superior survival in the short term. Based on these findings, NACT may be appropriate for select patients with advanced uterine serous carcinoma.

## Key Points

**Question:**

Is neoadjuvant chemotherapy associated with overall survival in women with metastatic endometrial cancer?

**Findings:**

In this cohort study of 4890 women with metastatic endometrial cancer, 952 women (19.5%) were treated with neoadjuvant chemotherapy. Survival for women treated with neoadjuvant chemotherapy was superior to that of women treated with primary debulking surgery for 3 to 8 months after initiation of treatment, after which time survival was superior for those treated with primary debulking surgery.

**Meaning:**

These findings suggest that women treated with primary debulking surgery are at increased risk of early death but have a more favorable long-term prognosis.

## Teaser

This cohort study uses data from the National Cancer Database to investigate the association of neoadjuvant therapy with overall survival in women with metastatic endometrial cancer.

## Introduction

Although endometrial cancer is often detected early and is associated with a favorable prognosis, approximately 10% to 15% of women present with metastatic disease beyond the pelvis and regional lymph nodes at the time of diagnosis.^1^ The prognosis for these women is poor, and these patients account for a disproportionate number of endometrial cancer–associated deaths.^2^ Five-year survival estimates for stage IV endometrial cancer range from 12% to 48%.^3^

Although prospective data are largely lacking and treatment is individualized, primary debulking surgery (PDS) is generally considered the criterion standard for the treatment of stage IV endometrial cancer.^4^ Prior studies have suggested that, similar to ovarian cancer, survival is dependent on the amount of residual disease after completion of surgery.^4^ In one series, survival improved from 6.7 months to 17.8 months for patients with optimal debulking.^5^ Given that cytoreductive surgery often requires radical resection of abdominal viscera, the morbidity associated with the procedure is substantial.^5^

For many solid tumors, use of neoadjuvant chemotherapy (NACT) before surgical resection has increased.^6,7,8,9^ A strategy of NACT allows for a more limited resection in responders and often decreases perioperative morbidity.^10^ For ovarian cancer, multiple randomized clinical trials have demonstrated that NACT and primary debulking are associated with similar survival, whereas NACT is accompanied by lower perioperative morbidity.^11,12^ To our knowledge, few studies to date have examined the efficacy of NACT for women with metastatic endometrial cancer.

The objectives of our analysis were 2-fold: first, to assess the trends in the use of NACT over time in women with stage IV endometrial cancer and second, to compare the survival of women treated with NACT with that of patients treated with primary surgical resection.

## Methods

### Data Source

We performed a retrospective cohort study using data from the National Cancer Database. The National Cancer Database is a joint project of the Commission on Cancer of the American College of Surgeons and the American Cancer Society. It contains data on patients with cancer from over 1500 Commission on Cancer–accredited hospitals, and it represents more than 70% of newly diagnosed cancer cases across the United States.^13^ The study analyzed deidentified data and was classified as nonhuman subjects research by the Columbia University Institutional Review Board. There was no patient contact and, therefore, informed consent was waived for participants. This study followed the Strengthening the Reporting of Observational Studies in Epidemiology (STROBE) reporting guideline.

### Patient Selection

We identified women who had stage IV malignant uterine cancer diagnosed as their only or first cancer with pathological confirmation from January 1, 2010, to December 31, 2015. The cohort was limited to women who were treated at the reporting hospital. We excluded women who were older than 70 years, who had neoadjuvant or intraoperative radiation therapy, for whom administration of chemotherapy was unknown or the time of chemotherapy administration was uncertain, and who had chemotherapy and surgery (hysterectomy or exenteration) initiated on the same day or more than 90 days after cancer diagnosis. To limit patients with significant comorbidity who may have had biased treatment decision-making, those who had a Charlson comorbidity score greater than 0 were also excluded as previously described.^14^

Patients were classified based on initial treatment approach. The NACT group included patients who had NACT followed by surgery and those who had initial NACT but did not undergo surgery. The PDS group included patients who underwent primary surgery as first treatment regardless of whether they received subsequent chemotherapy.

Patients’ demographic characteristics included age at diagnosis, race/ethnicity, year of diagnosis, insurance status, median household income in the patients’ zip code, and location based on the US Department of Agriculture Economic Research Service classification (Table 1). Tumor characteristics included tumor stage derived from the American Joint Committee on Cancer pathologic stage groups (stage IVA [tumor spread to the rectum or urinary bladder], stage IVB [distant spread of tumor], and stage IV NOS [not otherwise specified]), histology, and grade. Hospital characteristics included facility region and type as defined by the American Cancer Society’s Commission on Cancer Accreditation program (Table 1).

**Table 1.** Demographic and Clinical Characteristics of Patients, Crude and Inverse Probability of Treatment Weighted (Propensity Weighted)

Characteristic	Unadjusted			Propensity weighted		
	NACT, No. (%)		*P* value	NACT, No. (%)^a^		*P* value
	No	Yes		No	Yes	
All	3938 (80.5)	952 (19.5)	NA	3989 (77.5)	1159 (22.5)	NA
Age, y						
≤40	175 (4.4)	43 (4.5)	.26	178 (4.5)	57 (4.9)	.86
41-50	485 (12.3)	95 (10.0)		471 (11.8)	130 (11.2)	
51-60	1427 (36.2)	351 (36.9)		1450 (36.3)	415 (35.8)	
61-70	1851 (47.0)	463 (48.6)		1890 (47.4)	557 (48.1)	
Race/ethnicity						
White	2652 (67.3)	626 (65.8)	.79	2675 (67.1)	778 (67.2)	.99
Black	766 (19.5)	197 (20.7)		784 (19.6)	223 (19.2)	
Hispanic	262 (6.7)	70 (7.4)		272 (6.8)	78 (6.8)	
Other	214 (5.4)	50 (5.3)		215 (5.4)	66 (5.7)	
Unknown	44 (1.1)	NA^b^		44 (1.1)	13 (1.2)	
Year of diagnosis						
2010	555 (14.1)	106 (11.1)	<.001	540 (13.5)	152 (13.1)	.99
2011	638 (16.2)	123 (12.9)		620 (15.5)	183 (15.8)	
2012	648 (16.5)	138 (14.5)		641 (16.1)	182 (15.7)	
2013	713 (18.1)	160 (16.8)		713 (17.9)	213 (18.4)	
2014	670 (17.0)	201 (21.1)		710 (17.8)	210 (18.1)	
2015	714 (18.1)	224 (23.5)		764 (19.2)	219 (18.9)	
Insurance status						
Private	2179 (55.3)	507 (53.3)	.002	2189 (54.9)	644 (55.6)	.98
Medicare	1009 (25.6)	225 (23.6)		1006 (25.2)	289 (24.9)	
Medicaid	363 (9.2)	117 (12.3)		391 (9.8)	111 (9.5)	
Uninsured	256 (6.5)	54 (5.7)		253 (6.3)	69 (6.0)	
Other government/unknown	131 (3.3)	49 (5.1)		149 (3.7)	46 (4.0)	
Income, US$						
<38 000	697 (17.7)	184 (19.3)	.27	719 (18.0)	212 (18.3)	>.99
38 000-47 999	832 (21.1)	218 (22.9)		857 (21.5)	253 (21.8)	
48 000-62 999	1084 (27.5)	233 (24.5)		1075 (26.9)	308 (26.6)	
≥63 000	1308 (33.2)	314 (33.0)		1322 (33.1)	381 (32.9)	
Unknown	17 (0.4)	NA^b^		16 (0.4)	NA^b^	
Location						
Metropolitan	3283 (83.4)	821 (86.2)	.17	3350 (84.0)	979 (84.5)	.79
Urban	487 (12.4)	95 (10.0)		475 (11.9)	140 (12.1)	
Rural	68 (1.7)	16 (1.7)		68 (1.7)	17 (1.5)	
Unknown	100 (2.5)	20 (2.1)		96 (2.4)	23 (2.0)	
Facility type						
Academic/research	1939 (49.2)	522 (54.8)	.02	2012 (50.4)	599 (51.7)	.89
Community cancer	177 (4.5)	38 (4.0)		175 (4.4)	48 (4.1)	
Comprehensive community cancer	1358 (34.5)	297 (31.2)		1349 (33.8)	386 (33.3)	
Integrated network cancer	464 (11.8)	95 (10.0)		453 (11.4)	126 (10.9)	
Facility region						
Northeast	825 (20.9)	216 (22.7)	.23	847 (21.2)	246 (21.2)	.79
Midwest	988 (25.1)	254 (26.7)		1019 (25.6)	302 (26.1)	
South	1468 (37.3)	322 (33.8)		1460 (36.6)	433 (37.4)	
West	657 (16.7)	160 (16.8)		662 (16.6)	178 (15.4)	
Stage						
IVA	429 (10.9)	73 (7.7)	.01	410 (10.3)	122 (10.5)	.78
IVB	3134 (79.6)	788 (82.8)		3199 (80.2)	934 (80.6)	
IV NOS	375 (9.5)	91 (9.6)		380 (9.5)	103 (8.9)	
Histology						
Endometrioid	1114 (28.3)	223 (23.4)	<.001	1092 (27.4)	321 (27.7)	>.99
Serous	828 (21.0)	249 (26.2)		879 (22.0)	255 (22.0)	
Clear cell	117 (3.0)	34 (3.6)		123 (3.1)	34 (2.9)	
Carcinosarcoma	612 (15.5)	115 (12.1)		593 (14.9)	176 (15.2)	
Sarcoma	554 (14.1)	90 (9.5)		525 (13.2)	149 (12.9)	
EM NOS	614 (15.6)	188 (19.7)		653 (16.4)	188 (16.2)	
Other	99 (2.5)	53 (5.6)		124 (3.1)	36 (3.1)	
Grade						
Well differentiated	169 (4.3)	32 (3.4)	<.001	164 (4.1)	46 (4.0)	.99
Moderately differentiated	437 (11.1)	94 (9.9)		434 (10.9)	128 (11.0)	
Poorly differentiated	2451 (62.2)	471 (49.5)		2384 (59.8)	694 (59.9)	
Unknown	881 (22.4)	355 (37.3)		1008 (25.3)	291 (25.1)	

Abbreviations: EM, endometrial cancer; NA, not applicable; NACT, neoadjuvant chemotherapy; NOS, not otherwise specified.

^a^ Data represent the weighted cohort; after rounding to the nearest integer, the sum may not be 100%.

^b^ Cell size <10.

### Statistical Analysis

The trend of NACT use over time was plotted and examined using Cochran-Armitage trend tests. Frequency distributions between patient characteristics were compared using χ^2^ tests. To examine factors associated with NACT, we fitted a mixed-effect log-Poisson model adjusting for age, race/ethnicity, year of diagnosis, insurance status, income, location, facility type, region, stage, histology and grade, including hospital identification as a random effect for hospital-level clustering.

To better account for the conditional probability of treatment selection, we performed propensity score analysis with inverse probability of treatment weight (IPTW). We estimated each patient’s propensity to undergo NACT using a logistic regression model with the same set of covariates and calculated the IPTW as the reciprocal of the probability of receiving NACT.^15^ We stabilized the IPTW to reduce variability due to instability in estimation that could be induced by patients with very large weights.^16,17^ We used weighted contingency tables to assess the comparability of baseline characteristics. Patients diagnosed in 2015 were not included in the survival analysis because of missing survival data.

We performed an intention-to-treat (ITT) analysis that included all patients based on their initial treatment: chemotherapy for patients who underwent NACT and surgery in those patients in the primary surgery group. A second per-protocol (PP) analysis was undertaken in which the cohort was restricted to patients who received both treatment modalities: chemotherapy followed by surgery in the NACT group and surgery followed by chemotherapy in the primary surgery group.

All-cause mortality was compared using IPTW survival curves. The proportional hazards assumption was violated based on the crossing survival curves. Therefore, we estimated the hazard ratio (HR) of NACT for all-cause mortality using a flexible parametric survival model (a weighted Royston-Parmer model) to account for the time-varying hazards of NACT.^18^ The baseline survival function was estimated by a restricted cubic spline function with 3 *df*. The time-varying effect of NACT was accounted for using a second spline function with 2 *df*. The degrees of freedom (number of knots) were selected based on the Akaike information and Bayesian information criteria. Statistical analyses were performed from March 15, 2018, to July 20, 2018. All hypothesis testing was 2-sided, and *P* < .05 was considered statistically significant. All analyses were conducted using SAS software, version 9.4 (SAS Institute) and R, version 3.4.2 (R Foundation).

## Results

**Figure 1.**
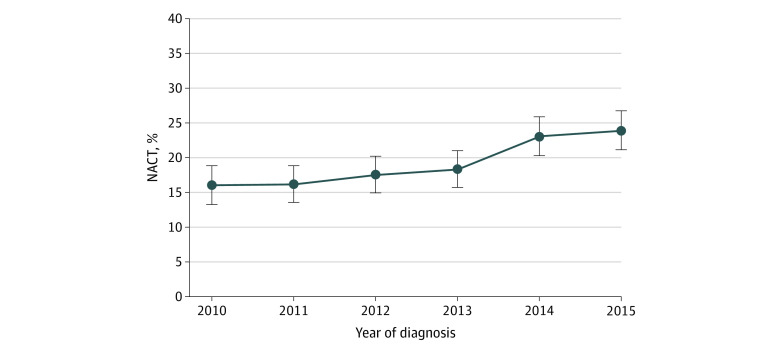
Percentage of Patients Receiving Neoadjuvant Chemotherapy (NACT) Over Time in the Crude Cohort Error bars indicate 95% CIs. *P* < .001 (Cochran-Armitage trend test).

A total of 4890 women (median age 60 years [interquartile range, 54-65 years]) with stage IV endometrial cancer were identified (eFigure in the Supplement). Within the cohort, NACT was used in 952 of 4890 patients (19.5%), whereas 3938 (80.5%) underwent primary surgery (Table 1). Use of NACT increased from 106 of 661 patients (16.0%; 95% CI, 13.2%-18.8%) in 2010 to 224 of 938 patients (23.9%; 95% CI, 21.2%-26.6%) in 2015 (*P* < .001) (Figure 1).

The clinical and demographic characteristics of the cohort are displayed in Table 1. In a multivariate model, patients with Medicaid were 25% more likely to receive NACT than women with private insurance (risk ratio [RR], 1.25; 95% CI, 1.01-1.55) (Table 2). Patients treated more recently were more likely to undergo NACT (RR for diagnosis in 2015 vs 2010, 1.42; 95% CI, 1.12-1.79), whereas women treated at urban centers were less likely to receive NACT than those treated at metropolitan hospitals (RR, 0.79; 95% CI, 0.63-0.99). Patients with stage IVB tumors were 31% more likely to undergo NACT than those with stage IVA neoplasms (RR, 1.31; 95% CI, 1.03-1.67). Similarly, women with serous carcinomas (RR, 1.38; 95% CI, 1.13-1.69) and other unspecified histologic subtypes (RR, 2.23; 95% CI, 1.64-3.05) were more likely to receive NACT than patients with endometrioid tumors.

**Table 2.** Multivariate Model^a^ for Factors Associated With Use of Neoadjuvant Chemotherapy

Characteristic	RR (95% CI)
Age, y	
≤40	1 [Reference]
41-50	0.85 (0.59-1.22)
51-60	0.98 (0.71-1.35)
61-70	1.01 (0.72-1.41)
Race/ethnicity	
White	1 [Reference]
Black	1.02 (0.85-1.21)
Hispanic	1.01 (0.78-1.32)
Other	0.99 (0.74-1.33)
Unknown	0.81 (0.41-1.57)
Year of diagnosis	
2010	1 [Reference]
2011	1.01 (0.77-1.31)
2012	1.08 (0.84-1.40)
2013	1.08 (0.85-1.39)
2014	1.37 (1.08-1.74)^b^
2015	1.42 (1.12-1.79)^b^
Insurance status	
Private	1 [Reference]
Medicare	0.90 (0.75-1.07)
Medicaid	1.25 (1.01-1.55)^b^
Uninsured	0.97 (0.72-1.30)
Other government/unknown	1.35 (0.98-1.86)
Income, US$	
<38 000	1 [Reference]
38 000-47 999	1.05 (0.86-1.29)
48 000-62 999	0.86 (0.70-1.06)
≥63 000	0.91 (0.74-1.12)
Unknown	0.97 (0.29-3.23)
Location	
Metropolitan	1 [Reference]
Urban	0.79 (0.63-0.99)^b^
Rural	0.95 (0.57-1.58)
Unknown	0.92 (0.57-1.47)
Facility type	
Academic/research	1 [Reference]
Community cancer	0.88 (0.62-1.23)
Comprehensive community cancer	0.90 (0.77-1.06)
Integrated network cancer	0.83 (0.66-1.05)
Facility region	
Northeast	1 [Reference]
Midwest	1.07 (0.87-1.31)
South	0.91 (0.75-1.12)
West	1.01 (0.80-1.27)
Stage	
IVA	1 [Reference]
IVB	1.31 (1.03-1.67)^b^
IV NOS	1.34 (0.98-1.85)
Histology	
Endometrioid	1 [Reference]
Serous	1.38 (1.13-1.69)^b^
Clear cell	1.25 (0.86-1.81)
Carcinosarcoma	0.89 (0.70-1.13)
Sarcoma	0.78 (0.61-1.01)
EM NOS	1.43 (1.17-1.75)^b^
Other	2.23 (1.64-3.05)^b^
Grade	
Well differentiated	1 [Reference]
Moderately differentiated	1.09 (0.72-1.63)
Poorly differentiated	0.88 (0.61-1.27)
Unknown	1.65 (1.13-2.39)^b^

Abbreviations: EM, endometrial cancer; NOS, not otherwise specified; RR, risk ratio.

^a^ Log-Poisson model was fitted in the crude cohort adjusted for age, race/ethnicity, year of diagnosis, insurance status, income, location, facility type, region, stage, histology, and grade. Hospital identification was included as a random effect to account for hospital-level clustering.

^b^*P* < .05.

**Figure 2.**
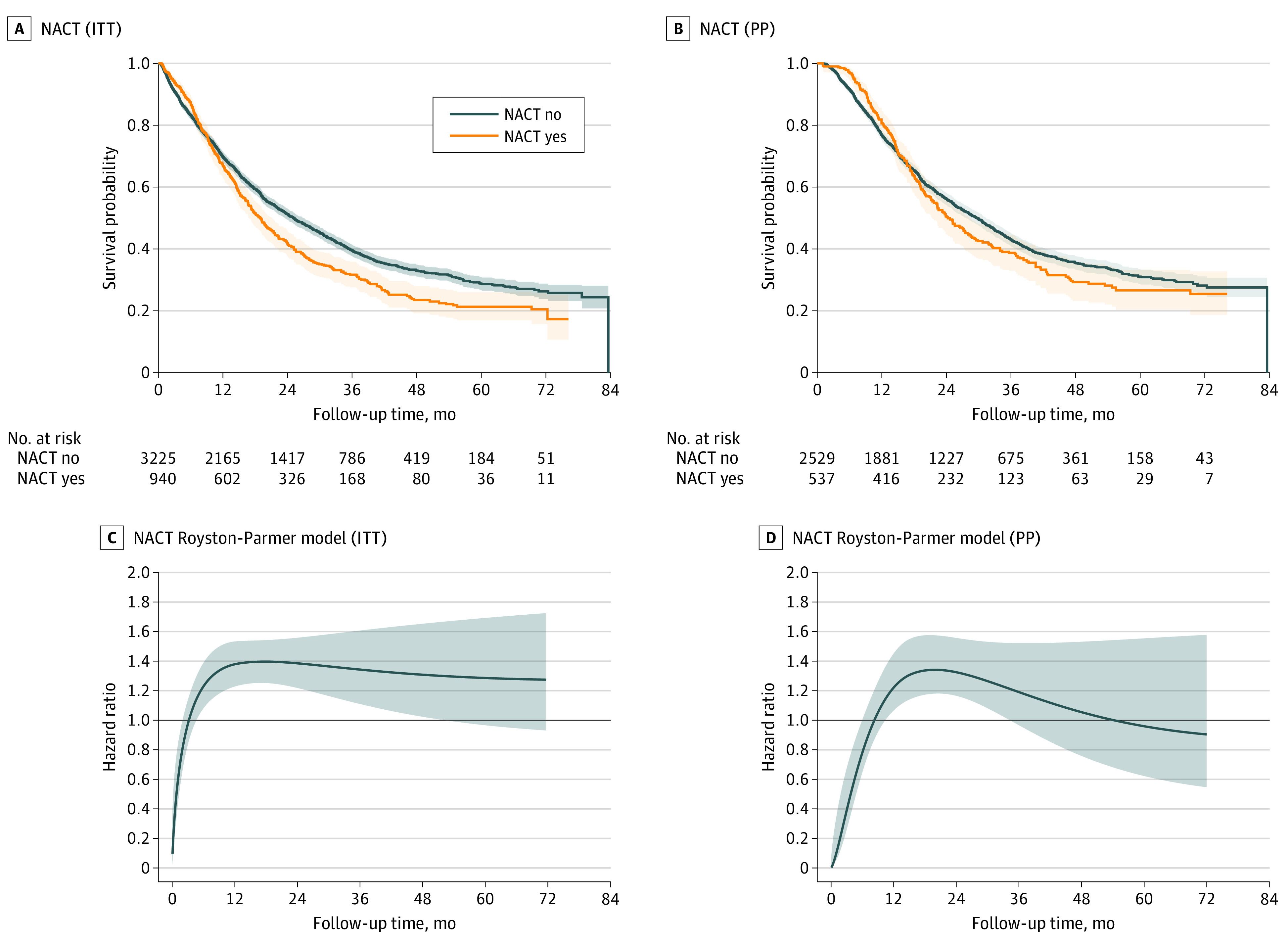
Inverse Probability of Treatment–Weighted Survival Curves Survival curves by A, NACT (ITT); B, NACT (PP); C, NACT Royston-Parmer model (ITT); and D, NACT Royston-Parmer model (PP). Shaded areas indicate 95% CIs. The bold horizontal line in panels C and D indicates a hazard ratio of 1.0. ITT indicates intention to treat; NACT, neoadjuvant chemotherapy; and PP, per protocol.

After propensity score weighting, the cohorts of women treated with primary surgery and NACT were well balanced (Table 1). In the ITT analysis of all patients, survival was superior for women treated with NACT for approximately the first 3 months after diagnosis, after which time the survival curves crossed and survival was superior for women who underwent primary surgery (Figure 2). A plot of the HR for mortality with use of NACT demonstrates an HR of 0.56 (95% CI, 0.39-0.80) in the first month after diagnosis, 0.81 (95% CI, 0.66-0.99) in the second month, similar HRs for months 3 and 4, and then an increased hazard of mortality from month 5 onward (HR, 1.17; 95% CI, 1.03-1.33) (Figure 2, Table 3).

**Table 3.** Propensity Score–Weighted Time-Varying Hazard Ratio of Treatment With Neoadjuvant Chemotherapy for All-Cause Mortality^a^

Follow-up time, mo	HR (95% CI)	
	ITT	Per protocol^b^
1	0.56 (0.39-0.80)	0.10 (0.03-0.32)
2	0.81 (0.66-0.99)	0.24 (0.12-0.50)
3	0.97 (0.83-1.14)	0.39 (0.24-0.65)
4	1.09 (0.95-1.24)	0.54 (0.37-0.78)
5	1.17 (1.03-1.33)	0.67 (0.50-0.88)
6	1.23 (1.09-1.39)	0.79 (0.63-0.98)
7	1.27 (1.13-1.44)	0.89 (0.74-1.07)
8	1.31 (1.16-1.48)	0.98 (0.83-1.16)
9	1.33 (1.18-1.50)	1.05 (0.90-1.24)
10	1.35 (1.20-1.52)	1.12 (0.96-1.31)
11	1.37 (1.22-1.53)	1.18 (1.01-1.37)
12	1.38 (1.23-1.54)	1.22 (1.04-1.43)
24	1.38 (1.23-1.56)	1.32 (1.14-1.53)
36	1.34 (1.12-1.61)	1.19 (0.95-1.50)
48	1.31 (1.03-1.66)	1.05 (0.74-1.51)
60	1.29 (0.97-1.70)	0.96 (0.60-1.53)
72	1.27 (0.94-1.73)	0.90 (0.53-1.56)

Abbreviations: HR, hazard ratio; ITT, intention to treat.

^a^ Royston-Parmer model was fitted in the inverse probability of treatment–weighted cohort. The baseline survival function was estimated by a restricted cubic spline function with *df* = 3, placing 2 knots at tertiles of the cumulative hazard distribution of all-cause death times and 2 knots at each boundary. The time-varying effect of NACT was estimated using a second spline function with *df* = 2.

^b^ Per-protocol analysis was limited to patients who had NACT followed by surgery vs patients who were treated with surgery primarily and had chemotherapy after surgery. Separate propensity score analysis was performed using the same methods as the main analysis. Patients diagnosed in 2015 were not included in the survival analysis.

A second PP analysis was performed just among women who actually received both surgery and chemotherapy. Among women who started treatment with surgery, 3139 of 3938 (79.7%) ultimately went on to receive chemotherapy. For those who initiated NACT, 555 of 952 (58.3%) underwent surgery. Similar survival trends were noted; however, in this analysis, mortality was lower for the NACT group until the survival curves crossed after approximately 8 months (Figure 2B). In this analysis, the HR for mortality was statistically significantly lower with receipt of NACT from months 1 to 6 after diagnosis and similar for NACT and primary surgery from months 7 to 10 after diagnosis, after which time the HR for mortality was higher among women treated with NACT (Figure 2D and Table 3).

## Discussion

The findings of this cohort study suggest that use of NACT for stage IV endometrial cancer has increased over time, and in 2015, nearly one-quarter of women with stage IV endometrial cancer received neoadjuvant therapy. Treatment with NACT was associated with decreased mortality immediately following initiation of therapy, but in women who were longer-term survivors, survival was superior for those who underwent primary surgery.

There are limited data describing the relationship between NACT and survival in patients with advanced-stage endometrial cancer.^19,20,21,22,23,24^ In a multicenter, retrospective review^19^ of 426 patients with stage IVB endometrial cancer, 125 (29%) underwent primary chemotherapy, and 59 of those women subsequently underwent surgery. Median overall survival was 21 months in women who underwent primary surgery vs 12 months in those treated with NACT. In addition, patients who underwent primary surgery had better performance status and fewer extra-abdominal metastases. Those women who underwent NACT and ultimately had surgery had survival times that were similar to the primary surgery group. Optimal debulking was achieved in 45% of the primary surgery group compared with 57% of the group who received NACT.^19^ In contrast, in a cohort of 34 women with stage IVB serous carcinomas, overall survival was 17 months in women treated with NACT vs 18 months in those who underwent primary surgery.^24^ Vandenput and colleagues^20^ prospectively examined NACT for stage IV endometrial cancer. Overall, 93% of patients responded to chemotherapy or had stable disease. Among women who underwent surgery, 80% had an optimal debulking. Median overall survival was 23 months.^20^ In a retrospective cohort of 102 patients who underwent NACT, complete debulking was achieved in 62% of women with endometrioid tumors and 56% of those with serous tumors treated with NACT. Median survival was 27 months for the cohort.^22^ In contrast, in a cohort of 39 patients who underwent NACT for advanced endometrial cancer, only 41% ultimately went on to surgery.^23^

These data highlight the fact that overall survival is poor regardless of the primary treatment modality for women with stage IV uterine cancer. The relationship between use of NACT and survival appears to be complex. In our cohort, results suggested that women treated with PDS were at increased risk of early death but had a more favorable long-term prognosis. We hypothesize that those patients who underwent PDS were at increased risk of early postoperative complications and mortality; however, those who tolerated the surgery had superior long-term outcomes. Patient selection for primary debulking among women with metastatic endometrial cancer is clearly of importance and warrants further study.

Potential benefits of NACT include decreased surgical morbidity and the early identification of disease resistant to treatment. A number of studies have reported that surgical morbidity is lower for women treated with NACT compared with PDS.^24^ In a cohort of women with stage IVB endometrial cancer, a strategy of NACT plus interval debulking surgery was associated with a statistically significantly shorter length of hospital stay (4 vs 6 days) and a trend toward shorter operative time with lower transfusion rates, with comparable rates of complete debulking.^24^ Similarly, Wilkinson-Ryan and coworkers^21^ reported that, compared with women who underwent primary debulking, those who underwent NACT had shorter mean operative times and shorter hospital stays, with similar rates of optimal debulking. These authors concluded that NACT may be appropriate for select patients with advanced uterine serous carcinoma.

To our knowledge, there is a paucity of data to date on the use of NACT for the early identification of chemotherapy-resistant disease and resultant patient outcomes. Hypothetically, information regarding response to initial treatment may inform decisions with respect to surgical intervention as well as earlier enrollment in clinical trials, palliative care referrals, and goals of care discussions that have favorable outcomes with regard to quality of life in end-stage disease.^25^ Further data exploring the predictive value of response rates to NACT as well as the benefits of NACT in patients felt to have unresectable disease at the time of diagnosis are certainly needed.

Although we noted that use of NACT was increasing overall, we identified a number of disparities in treatment allocation. Similar to institutional series, patients with serous tumors were more likely to receive NACT, which most likely reflects the aggressive nature of these neoplasms. Women with Medicaid were more likely to receive NACT. Medicaid recipients may have had more difficulty accessing care. Additionally, a number of studies have shown that both non-White women and underinsured women present with more advanced disease at the time of diagnosis.^26,27^

### Strengths and Limitations

Although our study benefits from the inclusion of a large number of patients, we acknowledge several important limitations. First, an important limitation in any study of neoadjuvant therapy is selection bias. We attempted to mitigate this bias through analysis using propensity score weighting. However, undoubtedly there remained some bias in the selection of patients with a more favorable prognosis for primary debulking. The decision to initiate chemotherapy vs undergo primary surgery is ultimately patient- and clinician-dependent and is based on numerous factors that cannot fully be characterized using medical records or observational data. Second, we limited our cohort to individuals with limited comorbidities in an attempt to mitigate selection bias of treating patients with poor performance status with NACT, as has been previously described.^28^ Although comorbidities may be a surrogate for performance status, we recognize that we lacked actual performance status and that these data may not be generalizable to all women with metastatic endometrial cancer. Third, we cannot exclude the possibility of misclassification of first line of treatment in a small number of women. Fourth, the National Cancer Database lacks data on recurrences and cause of death; thus, we were unable to analyze cause of death in our cohort. Finally, although the database captures women from a large number of hospitals, these data may not be representative of the entire population. However, to our knowledge, this is the largest analysis to date of use of NACT for advanced-stage endometrial cancer.

## Conclusion

In summary, the findings of this cohort study suggest that use of NACT is increasing in women with stage IV endometrial cancer. The relationship between use of NACT and survival is complex. In this study, women treated with PDS were at increased risk of early death but had a more favorable long-term prognosis. In contrast, women treated with NACT, particularly if they ultimately underwent surgery, had superior survival in the short term. These findings suggest that similar to its benefits in ovarian cancer, NACT could potentially lower perioperative morbidity and may serve as an important treatment option among women with metastatic endometrial cancer.

## Notes

Supplement. **eFigure.** Female Patients With Uterine Cancer, 2010-2015
