# Supplementary material for: Association of Neoadjuvant Chemotherapy With Overall Survival in Women With Metastatic Endometrial Cancer
Source: JAMA Netw Open. 2020 Dec 9;3(12):e2028612. doi: 10.1001/jamanetworkopen.2020.28612 (PMC7726635; doi:10.1001/jamanetworkopen.2020.28612)
Supplement: Supplement. — eFigure. Female Patients With Uterine Cancer, 2010-2015 [file jamanetwopen-e2028612-s001.pdf]

# Supplementary Online Content

Tobias CJ, Chen L, Melamed A, et al. Association of neoadjuvant chemotherapy with overall survival in women with metastatic endometrial cancer. *JAMA Netw Open*. 2020;3(12):e2028612. doi:10.1001/jamanetworkopen.2020.28612

**eFigure.** Female Patients With Uterine Cancer, 2010-2015

This supplementary material has been provided by the authors to give readers additional information about their work.

© 2020 Tobias CJ et al. *JAMA Network Open.*

**eFigure.** Female Patients With Uterine Cancer, 2010-2015

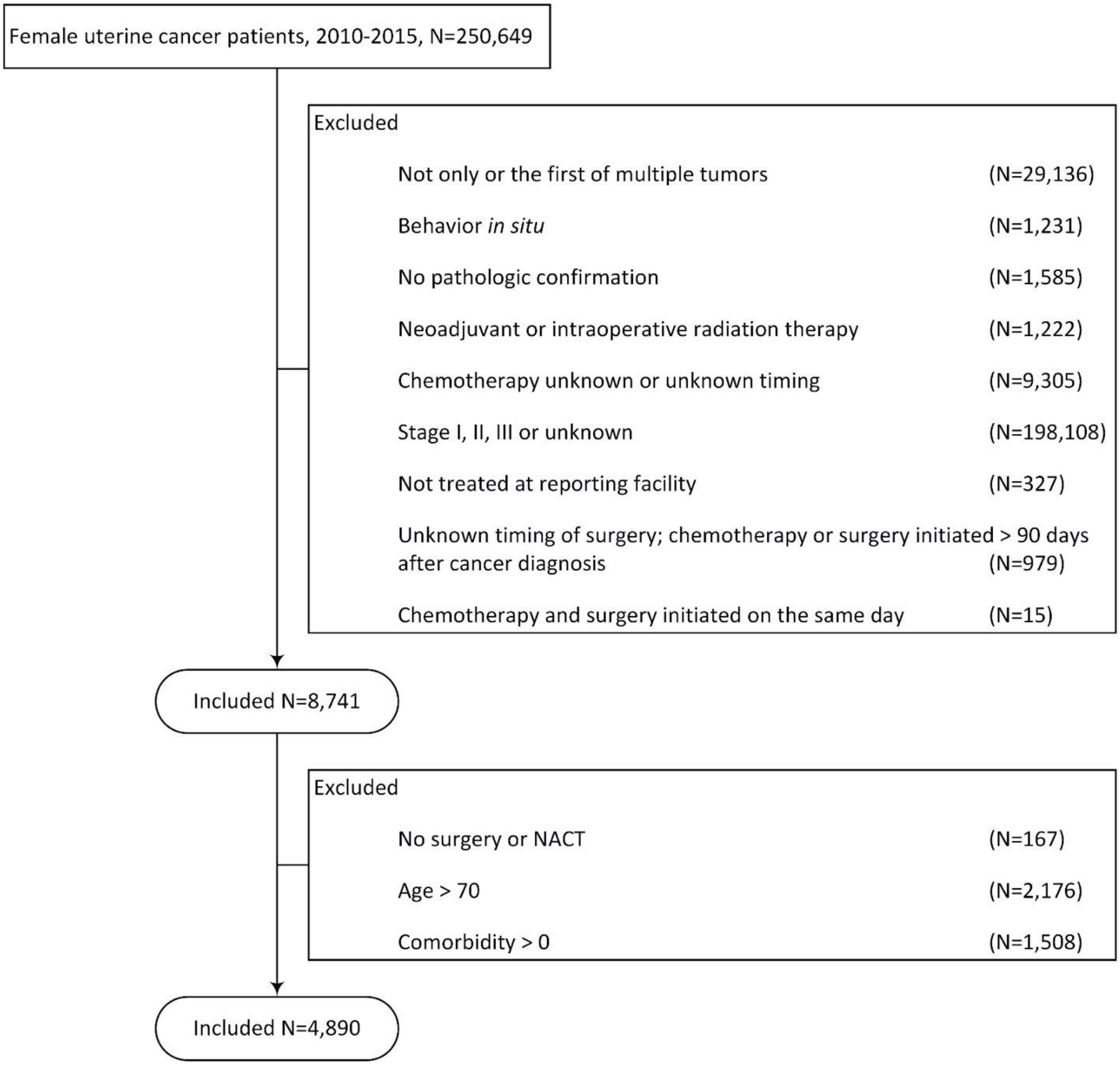


© 2020 Tobias CJ et al. *JAMA Network Open.*
